# Empowerment in the perioperative dialog

**DOI:** 10.1002/nop2.607

**Published:** 2020-08-18

**Authors:** Anna Abelsson, Peter Falk, Bengt Sundberg, Annette Nygårdh

**Affiliations:** ^1^ Department of Nursing Science School of Health Sciences Jönköping University Jönköping Sweden; ^2^ Surgical and Intensive care Clinics Värnamo County Hospital Region Jönköping county Sweden; ^3^ Surgical and Intensive care Clinics Ryhov County Hospital Region Jönköping county Sweden

**Keywords:** empowerment, nurse anaesthetist, patient, perioperative dialogue

## Abstract

**Aim:**

To describe how the nurse anaesthetist empowers the patient in the perioperative dialogue.

**Design:**

A qualitative descriptive design with interviews with 12 nurse anaesthetist (NA).

**Method:**

A hermeneutic text interpretation with a foundation in Gibson's empowerment model.

**Result:**

The results highlight Gibson's nursing domain: Helper, Supporter, Counsellor, Educator, Resource Consultant, Resource Mobilizer, Facilitator, Enabler and Advocate. The overall understanding is revealed as a relationship can be built through closeness between the patient and the NA. The NA helps the patient master the situation by talking to and touching the patient. The patient is helped to find their own strengths and to cope with their fears. The patients decide over their own bodies. When the patients do not want to or cope with protecting themselves, the NA protects and represents the patient.

## 1 INTRODUCTION

Empowerment means that the person can mobilize the resources necessary to take control of the situation (Gibson, 1991). Empowerment can be described as an inner strength that arises from a relationship with another human being. In a supportive relationship, the person can gain knowledge that generates positive empowerment within himself, which entails the possibility of self‐determination (Wåhlin, 2017). Empowerment is, therefore based on a mutual and secure relationship, through which people can develop strengths and strategies to manage their situation (Holmström & Röing, 2010). Gibson (1991) describes empowerment in health care as a process that includes both individual, and also interconnected behaviour patterns in both the patient and the nurse.

Previous research on empowerment in health care has included the midwife's work (Hermansson & Mårtensson, 2011), care in chronic diseases (Dowling, Murphy, Cooney, & Case y, 2011; Nygårdh, Wikby, Malm, & Ahlstrom, 2011), in psychiatric care (Ryles, 1999) and intensive care (Wåhlin, 2017). Many studies are quantitative and often related to patient education aimed at the management of the disease and for health promotion purposes. Foremost, empowerment is mostly described from the professional perspective and not from the patient perspective (Nygårdh et al., 2011).

This study focuses on empowerment in the perioperative context. The perioperative phase includes pre‐, intra‐ and postoperative care in connection with examinations and operative procedures with or without anaesthesia (Lindwall & von Post, 2009). During the perioperative phase, NA spends time with the patients and can create a relationship with the patient (Bredenhof Heijkenskjöld, Ekstedt, & Lindwall, 2010). The patient and NA can get to know each other (Pulkkinen, Junttila, & Lindwall, 2016). Strengthening the relationship is a common process between the patient and the NA (Castro, Van Regenmortel, Vanhaecht, Sermeus, & Van Hecke, 2016). This enables the patient to have confidence in the nurse. In the relationship, the patient needs to experience the feeling of being confirmed as a person in the vulnerable situation (Nygårdh et al., 2011). To be confirmed is to experience yourself as a unique human being (Nygårdh et al., 2011: Lindvall & von Post, 2013; Sundell, von Post, & Lindwall, 2010]. Trust and mutual respect are required for the interaction to be fruitful. In the relationship, they can both plan the care together (Lindwall & von Post, 2009). The patient is the main character in the patient–nurse meeting, where NA focuses on the patient and takes the time to listen to what the patient has to say (Nygårdh et al., 2011). This means the patient is an asset instead of a passive receiver of improved health (Castro et al., 2016; Gibson, 1991). The patient receives information and can ask questions that the NA can answer, thus alleviating the patient's anxiety (Sadati et al., 2013). Patients share their history with NA, which makes them feel safe when they hand over the control of their body in the hands of the NA (Lindwall & von Post, 2009). Patients have the confidence in NA to possess the competence to provide the best possible care (Nygårdh et al., 2011). Patients experience a sense of safety to have NA by their side during the perioperative care process (Papastavrou et al., 2012; Pulkkinen et al., 2016).

The opposite of empowerment is when the patient is met with non‐chalance and with no interest for the patient as a person. The patient becomes powerless and helpless and experiences a loss of control (Rappaport, 1984). When the NA is seen as a stranger, the patient experiences a lack of dialogue where the patient is not involved in the care decisions being made for him. Lack of dialogue can result in the feeling of threat to the patient's safety and identity in the care situation (Nygårdh et al., 2011).

Gibson (1991) has developed a model for visualizing empowerment based on three domains; (1) client domain, a domain that forms attributes that are derived from the patient. Examples of attributes in this domain are motivation, learning, and growth; (2) client–nurse interaction is a common domain that forms attributes that are derived from the partnership between the patient and the nurse. Examples of attributes in this domain are trust, empathy, and collaboration; and (3) the nursing domain, a domain that constitutes attributes that are derived from the nurse. In the nursing domains’ attribute, Gibson describes the beliefs that the nurse must hold and practice. The attributes describe that the individual's health is their own, and the healthcare professionals can promote health. The individual's capacity to develop must be respected as well as that the individual should be allowed to make his own decisions. Decisions should be based on information that healthcare professionals can provide the individual and thus support the individual to use their own resources. The individual should be seen as an equal in the relationship with the healthcare professional, and the individual's decision should be respected. It is a collaboration founded in trust that leads to empowerment. The attributes in this domain are Helper, Supporters, Counsellor, Educator, Resource consultant, Resource Mobilizer, Facilitator, Enabler and Advocate (Gibson, 1991).

The patient can experience the perioperative care as strange and unfamiliar. The NA needs to see and confirm the patient as a human to create a relationship and a trustful atmosphere. For in a safe care environment, NA empowers the patient to mobilize necessary resources to experience some form of control during the perioperative care.

### 1.1 Aim

One motivation for this study was to integrate Gibson's Empowerment Model (1991) and give the theory a meaning in perioperative practice. Another motivation was to gain insight into how the concept of empowerment is used by anaesthetist nurses in perioperative practice. This study aimed to describe how the nurse anaesthetist empowers the patient in the perioperative dialogue.

### 1.2 Design

The study had a qualitative design. Interviews were performed and analysed with hermeneutic text interpretation according to Lindwall, von Post, and Eriksson (2010) with a foundation in Gibson (1991) empowerment model.

### 1.3 Participants

In total, 12 nurse anaesthetists participated in the study; 7 males and 5 females, aged between 29–63 years (mean 52). A NA is defined as a Registered Nurse with postgraduate education in anaesthetist nursing. In Sweden, the NA has support from an anaesthesiologist bur otherwise independently induce and maintain general anaesthesia (National Association for Anesthesia & Intensive Care, 2008). The participants had an average of 13 years’ experience as NA (range 1–30 years). Inclusion criteria were NA willing to participate in an interview about empowerment. All participants were informed about the study by their department manager, and the personnel who were willing to participate contacted the researcher.

### 1.4 Data collection

The interviews were conducted in a private room at the participant's workplace with one participant at a time. The interviews all started with the same open‐ended question: Would you like to describe your perioperative meeting with the patient? To get a more in‐depth description from the participants, follow‐up questions like; Can you tell me more? How do you mean? were asked. A pilot interview was done to validate the questions. The interviews lasted from 15 to 20 min and were recorded and transcribed verbatim.

### 1.5 Ethical Consideration

This study followed anonymity and integrity in accordance with the ethical principles of the World Medical Association (2013). Ethical approval was not needed, according to Swedish law (SFS 2008: 192). Informed consent was obtained from each participant.

### 1.6 Data analysis

In accordance with the hermeneutic tradition, this data analysis is performed through understanding, interpretation and application (Gadamer, 1989). The hermeneutic text interpretation does not describe the person behind the text. Instead, the focus is on the meaning of the text. The basis for understanding the text is the researchers’ pre‐understanding, which can either enhance or cloud their view. According to Gadamer (1989), everyone has some form of existential pre‐understanding of life. But existential and professional pre‐understanding is not the same and should therefore not be treated as equal. According to Lindwall et al. (2010), the researchers’ pre‐understanding has risen from the individual's values and prejudices. It is a pre‐understanding arising from the nursing profession, which the nurse acquires through the culture they take part in. A culture that can be seemingly obvious but that the individual is not always aware of since it is incorporated as if being part of their being (Polanyi, 1966). The hermeneutic design helped us discover both unknown but also already known patterns in perioperative practice (Lindwall et al., 2010). The text was interpreted in the following five steps:

#### 1.6.1 Integrating the text with the reader

The text is treated as an original source, and in the critical examination, the text relevance to reality is the foundation of validity (Lindwall et al., 2010). In the first reading, the text was read from the beginning to the end. The text spoke to the researchers as to a NA in the care of a patient in the perioperative context. The researchers' professional pre‐understanding made the text understandable (Lindwall et al., 2010).

#### 1.6.2 Fusion of horizons

The reality of the text becomes part of the researcher in the dialogue with the text (Lindwall et al., 2010). Through the fusion of horizons, it revealed how nurse anaesthetists experienced the perioperative dialogue with the patient.

#### 1.6.3 Putting new questions to the text

From the text, questions were generated: Which actions, impacting patients, require empowerment? The answers to the questions were significant expressions and quotations with common and distinguishing qualities.

#### 1.6.4 Summarizing the meaning units in the themes

The meaning units were read in search of the common quality of the significant expressions (Lindwall et al., 2010). Meaning‐unit by meaning‐unit, the text answering to the *Nursing domain* in Gibson's Empowerment model, (1991) was placed into the domains nine themes: *Helper, Supporter, Counsellor, Educators, Resource Consultant, Resource Mobilizer, Facilitator, Enabler* and *Advocate*. Quotes from the original texts were used to describe each theme.

#### 1.6.5 New understanding

The themes were confirmed through a comparison with the text, and a new understanding was searched for. The processing of the data was an ongoing and reflexive process from the parts to the whole and from the whole to the parts, according to the hermeneutic circle. Through the circle, a new understanding is formed, which is valid and free from inner contradictions (Lindwall et al., 2010).

### 1.7 Validity and reliability

The strategies for ensuring rigour in the study have been our choice of research design and the appropriateness of the method to answer the research questions. The method has carefully and thoroughly been used to ensure the accuracy of the results due to the potential of subjectivity inherent in qualitative research. To ensure validity and reliability, both authors worked with the texts to ensure that the results accurately reflect the data. Through peer debriefing, the researcher was able to improve the validity by reducing the risk of individual methodological bias. The reliability was ensured through the consistency of the analytical procedures achieved by two researchers. This included personal biases that otherwise may influence the results (Cypress, 2017). The application of this study's findings to other contexts is questionable due to its very specific context in accordance with Noble and Smith (2015).

## 2 FINDINGS

The result is presented according to Gibson’s (1991) nursing domain, which describes how the NA empowers the patient in the perioperative dialogue in the role as a *Helper, Supporter, Counsellor, Educator, Resource Consultant, Resource Mobilizer, Facilitator, Enabler* and *Advocate*.

### 2.1 Helper—Helping the patient to take control of the situation

The NA described how they, in the perioperative dialogue, helped the patient to master the patient's situation. Through physical contact between the NA and the patient, the NA perceived the patients’ feeling being able to cope more easily with their vulnerable situation. Physical contact during the conversation helped the patient to cope with negative emotions and thoughts in the face of impending anaesthesia.

*You try to talk to the patient while holding their hand or touching their arm*.

To distract the patient was described as a way to help the patient to master their situation. In the perioperative dialogue, focusing on the patient's experiences and needs is in contrast to patient's fears, resulting in a positive impact on the patient's ability to cope with the situation.

In the role of the Helper, the NA described how the perioperative dialogue helped the patient master situations perceived as negative or frightening. They use conversations with the patient, which could be intensified by touching the patient.

### 2.2 Supporter‐ —Create safety and involvement

The physical closeness that existed between the patient and the NA was described as facilitating the creation of safety for the patient in the perioperative dialogue. Being close to the patient meant to sit next to the patient, to touch the patient and to speak calming.

*I can stroke the patient on the cheek and say: I am sitting here right next to you. I see and hear you at all time*.

As the care was given in physical closeness between the patient and the NA, they both confirmed each other through eye contact and body language. The confirmation improved the possibility of a relationship and a dialogue between the patient and the NA.

NA created safety in the perioperative dialogue by being physically close to the patient. The proximity makes it easier to build a relationship with the patient. The proximity also allowed the NA to confirm the patient both verbally and non‐verbally. The patient was involved in the care through a dialogue with the NA. Participation demystified the anaesthesia and reduced the experience of exposure of the patient.

### 2.3 Counsellor—Give advice and show the path

In the role of an adviser in the perioperative dialogue, the NA was a guide based on the patient's needs and desires. To show the way, was to show without taking over the controls from the patient.

*I want to know how the patient feels and that it feels ok during the anesthesia. If the patient can be comfortable and not show signs of panic in their eyes*.

NA's experience and competence in perioperative work enabled them to guide the dialogue and adapt it based on the patient's state of mind. When the dialogue was based on the patient's needs, the NA felt that it created confidence in the patients.

*You gain experience in being able to create a connection with the patient. To listen to what the patient says and take them seriously*.

To be a guide meant that, in the perioperative dialogue, guiding the patient and let them make the decision. The NA profession included knowledge and experience that gave an understanding of the patient's needs and wishes. The professionalism of the perioperative dialogue created the experience of safety in the patient.

## 3 Educator—Promote well‐being

Before the anaesthesia, the NA educated the patient about the events before, during and after the anaesthesia. The fears that the patient might experience, often based on prejudice and misconceptions, could be eliminated. The NA described how educating the patient helped in coping with the fear of losing control during anaesthesia.

*When you inform the patient, they can ask about issues they have read in a newspaper. Such as patients waking up during the anesthesia or patients being very anxious about anesthesia. This is mostly about losing control of oneself*

When the patient could learn about their environment and the medications that would be used, the patient became more involved in the care process. The patient gained an understanding of the impact of the medications on the body to a greater extent and could then experience increased well‐being before anaesthesia.

*We're talking about anesthesia and what's going to happen—explaining which drugs are being administered and how the drugs work. Then you show the respiratory mask and the patient can try to breathe in it. Often you have to repeat yourself*.

Educating the patient in the perioperative dialogue gives the patient the opportunity to manage their fears. When the patient was educated, participation increased, resulting in increased well‐being.

### 3.1 Resource consultant‐ —Assist the patient in finding their own strength

In the role of a resource consultant, the NA described themselves as assistants in the patient's search for their own strengths and resources. By assisting the patient in finding their own strength, the patient could better cope with the situation they were in.

*Patients are anxious and fearful of different things of the anesthesia. Using your experience, you can find out how to get in touch with all the different patients, young women who cry or older gentlemen who are angry*

To adapt to the patient's needs, it was assumed that the patient trusted the NA's competency level and felt safe. Without safety, the patients could not find their own strength.

*In the conversation with the patient, you demonstrate that you are competent. The patient becomes more confident when they learn that you know what you are doing and that you have done this many time before*.

In the perioperative dialogue, NA helped patients find their own strength to cope with the situation. For the patient to find their own strength assumes that there is confidence in the NA. A positive anaesthesia experience resulted in a strength to handle future anaesthesia.

### 3.2 Resource mobilizer—Letting the patient take the command

In the role of resource mobilizer, the patient could decide to what degree they wanted to be involved in their care. The NA described how they could help the patient to take command and participating in their own care if they chose to.

*I ask my questions and listen to the answers. You get to identify the patient who wants to have short concrete answers or the patient who wants information to keep track of everything. One patient would prefer to be taken care of and the other has everything explained to them. That's how you do it*.

To help the patient take command was grounded in good communication. The communication helped the patient to use their own strength and their own resources. The communication needed to be in such a way that the patient had control over their choices.

*You ask if they want you to talk and tell them what you are doing. If I am present in the moment and try to adapt to both what the patient says, but also to what they radiate physically, I can meet the patient's wishes*.

The NA in the perioperative dialogue allowed the patient to take the command. The patient gets to influence and participate in their care based on their conditions and resources. In communication, the patients expressed their thoughts and needs, which the NA could respond to.

### 3.3 Facilitator—Create a relationship

Being honestly interested and daring to participate in the conversation was a precondition for the patient to have confidence in the NA. The NA described that if they did not show honesty in the conversation or did not dare to participate in the difficult conversations, the patient quickly lost confidence in them.

*Patients gain confidence in me in a matter of seconds if I really meet the patient. It is really important that they feel honesty from me. That I am interested in them. That I am in favor of this being important for them. I must never be afraid or avoid the patient when I meet them*.

Patients having confidence in the NA enabled the possibility of a relationship between the patient and the NA. In a working relationship, the patient could express their desires and needs to NA. They felt that they were available to the patient who trusted them.

*I need to have the patient's confidence during the anesthesia. For that, the patient needs to know who I am and be able to trust that I take responsibility for them during the anesthesia. If you establish good communication, they often feel safe. They will tell you if something is bothering them*.

The patient had confidence in the NA when the NA was honestly interested and involved in the perioperative dialogue. Trust was the prerequisite for a relationship between the patient and the NA. The relationship enabled wordless communication between the patient and the NA.

### 3.4 Enabler—Enabling participation

In the role of the enabler, NA could facilitate the participation of the patient. This way, the patient could influence their care. Participation was based on the patient being informed of different alternatives, without the encouragement of any specific choice.

*I give the patient the opportunity to decide. First, I inform about both the possibility of being awake and sedated. Then the patient decides, and then I usually tell them that if they change their mind, we can make a new decision*.

When the patient is involved in the perioperative dialogue, it is the patient's preference that controls the environment and medical administration to some extent. The patient can choose to be awake or to sleep for a while. To be involved in the care during anaesthesia leads to the patient maintaining their human dignity.

### 3.5 Advocate—Protect and represent the patient

The NA described that they were in the operating room for the patient's sake. The NAs were there for the patient to have a good experience of the anaesthesia. When the patient lost control of the situation or chose not to be involved in the care, the NA described an obligation to represent the patient and to protect the patient. To perform care actions with good intentions, for example, during an emergency situation, where talking to the patient were neglected in order to sedate them quickly, could still be considered by the NA as abusive of the patient.

*It is a big dilemma where you can feel that you really have to deviate from one's own purpose with the meeting with the patient. It's the most urgent caesarian, where we only take the patient, put on the mask, and sedate. One might call this a borderline positive abuse*.

Ethical conflicts were often experienced in emergencies but could also occur at other times. When the operator found something serious during the operation, and the NA considered that the patient was not susceptible to information at the time, there was a conflict in being the representative of the patient.

*Yes, I tell the patient that the surgery has gone well, as you do at all times I think, even if the result of the operation is not good. In this particular situation, the patient is not receptive to any other information, so you try to maintain the patient's feeling of safety*.

The NA advocated for the patient in the perioperative dialogue. To advocate for the patient was to inform and give the patient a good experience of the anaesthesia. When the patient lost control or did not want to be in control of the situation, the NA advocated for the patient. There was an ethical conflict in being the patient's advocate that involved protecting the patient in a vulnerable situation but still provide the best care for the patient.

## 4 DISCUSSION

The concept Empowerment refers to coping with difficulties and conquering the feeling of helplessness. In this study, we chose to use the empowerment model for nursing by Gibson (1991) as a grid for NA stories. The result is therefore based on the nine themes; *Helper, Supporters, Counsellor, Educator, Resource Consultant, Resource Mobilizer, Facilitator, Enabler* and *Advocate* in the nursing domain in Gibson's empowerment model (1991).

The results showed how the NA is trying to help the patient to master the powerlessness that the patient is in. Empowerment is described as a useful concept in different context when the patient experiences powerlessness (Slatyer, Williams, & Michael, 2015; Wåhlin, 2017). Powerlessness can mean thoughts of life and death, but also a tacit desire to feel like a whole human being with dignity, during the perioperative phase (Pulkkinen et al., 2016). The basic driving force of empowerment is the patient's desire or the courage to surrender to the inevitable that anaesthesia involves (Liebenhagen & Forsberg, 2013).

In the role of the Helper, the NA helps the patient master the situation. The patient tries to maintain control, however, must surrender into the hands of the NA during the anaesthesia. As an educator, the NA can help the patient cope with fears that existed before the anaesthesia. Fears previously described in connection with anaesthesia are never regaining consciousness again. To be semiconscious during surgery because of insufficient anaesthesia is also a fear that patients describe. To preserve their dignity during the perioperative period, the NA needs to understand the patient's experience of loss of control (Liebenhagen & Forsberg, 2013). When NA meets the patient with kindness and genuine interest (Nygårdh, Malm, Wikby, & Ahlström, 2012), the patient is seen as a unique human being where they are also allowed to participate in their own care (Lévinas, 1985). To be heard and seen is to be recognized and confirmed as a human being (Wåhlin, 2017).

The NA is a Supporter by being physically close to the patient, which facilitates establishing a relationship with the patient. Being close means that the NA can support the patient, both verbally and non‐verbally. The physical proximity that is described between the patient and the NA facilitated the creation of safety and participation of the patient in the perioperative period. The proximity to NA is both a physical but also an emotional closeness throughout the perioperative process. Since NA is not always in the patient's sight, a physical presence, such as touching the patient, can give a feeling of safety, especially in waking the patient (Lindwall, von Post, & Bergbom, 2003). It leads to the patient never experiencing physical or mental loneliness or omission (Arakelian, Swenne, Lindberg, Rudolfsson, & von Vogelsang, 2016; Karlsson, Ekebergh, Larsson Mauleon, & Almerud Österberg, 2013). The closeness also allows the NA to get to know the person behind the patient as someone with a will, emotions and needs (Ekman et al., 2011). In the role of the Resource Consultant, the NA helped the patient find his own strength to cope with the situation. It empowered the patient to feel safe in the care and trusted the NA as competent and professional. Without feeling safe, the patient could not find his or her own strength. Previous research shows that patients can experience confidence in the nurse (Nygårdh et al., 2012). Trust and a mutual and reliable relationship, where the patient can find a sense of inner strength leads to empowerment (Wåhlin, 2017). Because when the patient gets help to mobilize and improve their own resources, it enables them to take control of their own lives (Gibson, 1991). In the role of Resource mobilizer, the NA helps the patient to take command of their situation. The patients participate in their care based on their own conditions and resources. Wåhlin, (2017) describes how information needs to be adapted to the patient's needs and to the patient's ability to process the information. The NA should always have the intention to protect and represent the patient in his or her vulnerable situation (Sundqvist, Nilsson, Holmefur, & Anderzén‐Carlsson, 2018).

With NA as the Facilitator, the patient has confidence in the NA. The honest interest and participation in the perioperative dialogue is the prerequisite for a relationship between the patient and the NA. The relationship enables non‐verbal communication to occur between the patient and the NA. In the Enabler role, the patient is involved in the care and the patient's wishes control the circumstances and the medical administration. When the patients can control the position of their body at anaesthesia, the patient retains his or her human dignity. But in emergency care situations, sedating the patient quickly could be perceived as an abuse of the patient because of the lack of information or involvement of the patient. Representing the patient could sometimes result in an ethical conflict over what was best for the patient. This experience of powerlessness in the NA has previously been described by Slatyer et al. (2015) as non‐empowerment. When the NA cannot help the patient, the NA experiences psychosocial stress and powerlessness, which can have a negative effect on NA's self‐esteem. Ethical conflicts were often experienced in emergencies but could also occur at other times. In acute situations, there was no time for providing information, neither enabling the patient's participation. The NA made decisions for the patient's well‐being.

To advocate for the patient means that the NA protects and represents the patient when the patient cannot, wants to or is not able to protect himself. NA described that they were there for the patient. When the patient lost control of the situation or chose not to be involved in the care, NA described an obligation to advocate, protect and represent the patient. As previously described, there is a clear need to both protect and represent the patient in the perioperative care environment where the patient loses control (Nilsson, 2013). By speaking on behalf of the patient, NA acts as the patient's bodily extension (Karlsson, Ekebergh, Larsson, & Almerud Osterberg, 2012; Sundqvist, Holmefur, Nilsson, & Anderzen‐ Carlsson, 2016). The NA shows concern and respect for the patient's integrity and dignity (Munday, Kynoch, & Hines, 2015). By their presence, the NA is responsible for the patient's emotional and physical safety (Schreiber & Macdonald, 2010; Shannon, 2016). The NA also regard themselves as a protector who acts to protect the patient's body against injury (Sundqvist & Carlsson, 2014). They, therefore, want to place the patient's body in positions to prevent injuries (Nilsson, 2013).

## 5 CONCLUSION

This study shows how a relationship can be created through closeness between the patient and the NA. With the help from the NA, the patient can use their own strengths and master the situation. The NA empowers the patient who is treated as an equal with the right to self‐determination. The patient decides over his or her body, which means that the patient retains his or her dignity. With empowerment in the perioperative dialogue, the patient experiences a trustful atmosphere where the patient and the NA can meet. The NA shows an honest interest and listens to the patient's wishes, which empower the patient to trust the NA to represent them when they cannot, does not want to or is not able to protect themself.

## 6 CONFLICT OF INTEREST

The authors declare that there is no conflict of interest.

## Data Availability

All data generated or analysed during this study are included in this published article.
